# Predictive Factors of Outcomes in Acute Subdural Hematoma Evacuation

**DOI:** 10.7759/cureus.31635

**Published:** 2022-11-18

**Authors:** Zainab Manan, Shafique-ur Rehman, Abdul Aziz Khan, Syed Faizan Hassan Shah, Idress Ahmed, Mehboob Khan

**Affiliations:** 1 Neurosurgery, Ayub Teaching Hospital, Abbottabad, PAK; 2 Neurological Surgery, Hayatabad Medical Complex, Peshawar, PAK; 3 Surgery, Queen Elizabeth Hospital Birmingham, Birmingham, GBR

**Keywords:** asdh factors, traumatic asdh, asdh outcome, asdh association, acute subdural hematoma

## Abstract

Objective: This comparative cross-sectional study was conducted in the Departments of Trauma and Neurosurgery, Ayub Teaching Hospital, Abbottabad, Pakistan from September 2021 to February 2022 to study predictive factors of outcomes in acute subdural hematoma evacuation.

Methodology: A total of 101 patients with confirmed diagnosis of acute subdural hematoma (ASDH) who underwent surgical evacuation by consultant neurosurgeon were included in the study. A detailed clinical proforma was designed to document all the clinical and demographic details of these patients at the time of admission. Glasgow Coma Scale outcome score (GOS) was used to assess the outcome of patients after the surgery. Sociodemographic and clinical parameters were associated with outcome of surgery in our study participants.

Results: Out of 101 patients, 55 (54.5%) were males and 46 (45.5%) were females. Mean age was 43.66±19.66 years with 7.39 as mean Glasgow Coma Scale (GCS) at presentation. Road traffic accident (RTA) 62 (61.4%) was most frequent mechanism of injury followed by fall from height (19.8%) and history of assault (13.9%). In our study, 59 patients had poor outcomes while 42 had good outcomes. Elder age, low GCS at presentation, and use of oral anticoagulant were associated with poor outcomes while pupillary reaction had no effect on the outcome after application of test of significance.

Conclusion: More than half of the patients managed with surgical evacuation for acute subdural hematoma as per guidelines at our neurosurgical unit had poor outcomes according to Glasgow Coma Scale. In this study, advancing age (>60 years), low GCS score at presentation, and use of oral anticoagulation therapy emerged as significant risk factors for poor outcomes in participants. Pupillary reaction had no effect on outcomes as per this study but this needs further evaluation in future studies.

## Introduction

Trauma is one of the leading causes of mortality and morbidity, especially among patients of younger age group [1]. Head trauma may lead to multiple intracranial and extracranial manifestations [2]. Acute subdural hematoma (ASDH) is one of the commonest neurosurgical emergencies encountered by trauma and neurosurgeons across the globe [3]. Timely detection and surgical intervention may save the patient from otherwise grave consequences of this clinical condition.

Surgical evacuation is primary mode of management in patients who present with acute subdural hematoma [4]. Studies across the globe show that overall success rate of this procedure is good but still a number of demographic and clinical factors may affect the outcome of this procedure in a number of ways [5]. Surgeons in various settings have developed protocols to improve the outcome of this surgical procedure [6].

Studies have been done in various parts of the world to look for outcomes of surgical procedures in patients with acute subdural hematoma. Dijck et al. in 2019 assessed outcomes in patients who were operated for acute subdural hematoma [7]. They also looked for cost-benefit analyses for these procedures. It was concluded that outcomes in most cases was unfavorable and dependent on Glasgow Coma Scale (GCS) score at presentation. They critically analyzed the value-based cost-effectiveness of surgical treatment of patients with traumatic acute subdural hematoma. Tverdal et al.* *in 2022 studied neurosurgical emergency procedures at their center and came up with the findings that patients with old age and low GCS at time of injury were more at risk of poor outcomes [8]. Portuguese patients were studied in 2018 for predictive factors of poor outcomes in patients who underwent evacuation of acute subdural hematoma [9]. They concluded that low GCS at admission, spontaneous etiology, and craniectomy were associated with the worst outcomes in their study participants.

Neurosurgical facilities are evolving in our part of the world. Trauma surgery does not exist as a separate specialty in Pakistan therefore most of the burden of head injury is exclusively dealt by neurosurgeons. A recent local study captured data from 30 patients of acute subdural hematoma surgeries. It was revealed that still it is a potentially fatal condition in our part of the world. Early diagnosis and aggressive post-operative management in critical care units may reduce the mortality related to this condition [10]. Limited local data has been available regarding factors that predict poor outcomes in these patients. We therefore conducted this study with the rationale to study predictive factors of four outcomes in acute subdural hematoma evacuation performed at Ayub Teaching Hospital, Abbottabad.

## Materials and methods

This comparative cross-sectional study was conducted in the Departments of Trauma and Neurosurgery, Ayub Teaching Hospital from September 2021 to February 2022. The written informed consent was taken from the patient’s parents. Sample size was calculated by using the WHO sample size calculator by keeping population prevalence proportion of poor outcomes in patients with acute subdural hematoma at 78% and the margin of error at 10% [11]. Primary data was collected via non-probability purposive sampling technique.

Inclusion and exclusion criteria

The study included all patients of both genders (18-85 years) who were confirmed to have acute subdural hematoma on CT scan brain by a consultant radiologist and neurosurgeon. Patients with any malignant conditions or autoimmune disorders were excluded from the study. Those who, or their caregivers, refused surgery or were transferred to another public or private facility were excluded from the study. Patients who required multiple surgeries at the time of presentation were excluded as well.

After the written informed consent of parents, pediatric patients who fulfilled the above-mentioned inclusion criteria were included in the study. Acute subdural hematoma was diagnosed by consultant neurosurgeon on the basis of relevant clinical and neuroradiological findings [12]. All of these patients underwent baseline investigations and were prepared for emergency surgery in liaison with anesthetist. The consultant neurosurgeon who was on duty at the time of presentation performed evacuation surgery via set protocols [13]. Outcomes of patients were assessed by Glasgow Outcome Scale (GOS) with a range of 1-3 graded as poor outcome and a range of 4-5 as good outcome [14]. GOS score was calculated at the time of discharge from hospital.

A descriptive statistical analysis was carried out with SPSS version 23.0 (Armonk, NY: IBM Corp.). Mean and standard deviation was calculated for age of patients. Frequency along with percentage was calculated for gender and other demographic and outcome variables. Pearson's chi-square test was used to assess association between dependent and independent variables by keeping the p-value≤0.05 as significant.

## Results

This study was conducted on 101 subdural hematoma patients. Out of these, 55 (54.5%) were males and 46 (45.5%) were females. Mean age of patients was 43.66±19.66 years. Table 1 shows basic demographics, neurological status, mechanism of injury, and co-morbidities of study sample along with outcomes. Mean GCS at presentation was 7.39 with 11/15 being the most frequent. Overall, road traffic accident (RTA) 62 (61.4%) was the most frequent mechanism of injury followed by fall from height (19.8%) and history of assault (13.9%). Most patients (47.5%) had no co-morbid condition, however, 24.8% had cardiac problems, 19.8% had thrombosis, and 7.9% reported other comorbidities. Overall 42 (41.6%) had good outcomes while 59 (58.4%) had poor outcomes.

**Table 1:** Basic demographic profile of study population. GCS: Glasgow Coma Scale; RTA: road traffic accident

Parameters		Frequency (N)	Percentage (%)	Mean±SD
Age	<60 years	65	64.4%	43.33±19.66
	>60 years	36	35.6%	
GCS at presentation	>8/15	55	54.5%	7.39±2.28
	<8/15	46	45.5%	
Gender	Males	55	54.5%	-
	Females	46	45.5%	
Mech of injury	RTA	62	61.4%	-
	Assault	14	13.9%	
	Fall	20	19.8%	
	Others	5	5%	
Co-morbidity	Nil	48	47.5%	-
	Cardiac	25	24.8%	
	Thrombosis	20	19.8%	
	Others	8	7.9%	
Glasgow Outcome Score	Poor outcome	59	58.4%	-
	Good outcome	42	41.6%	

Table 2 shows the factors studied for association with outcomes of ASDH based on the Glasgow Outcome Scale. Pearson's chi-square analysis shows statistically significant evidence of association of age, GCS at presentation, and oral anticoagulant therapy with outcomes while pupillary reaction has no statistically significant effect on outcomes.

**Table 2:** Factors associated with poor outcomes in our study participants. GCS: Glasgow Coma Scale

Determinant	Category	Frequency (N)	Percentage (%)	p-Value
GCS at presentation	>8/15	55	54.5	0.004
	<8/15	46	45.5	
Age group	<60 years	65	64.4	<0.001
	>60 years	36	35.6	
Oral anticoagulant therapy	Not taking	59	58.4	<0.001
	Taking	42	41.6	
Pupillary reaction	Anisocoric/non-reactive	63	62.4	0.30
	Isochoric/reactive	38	37.6	

## Discussion

Results of this study showed that outcomes of evacuation procedure for acute subdural hematoma is not very promising in our data set and is dependent on certain factors. Traumatic injuries have been on rise and head trauma-prone individuals are towards acute subdural hematoma which may become life-threatening if not managed in time. Limited neurosurgical services are available in our country and that too mostly in big teaching setups. Enough epidemiological data has not been available with regard to the outcomes of these patients in Pakistan, especially from Khyber Pakhtunkhwa province. We conducted this study with the aim to study predictive factors for outcomes in acute subdural hematoma evacuation performed at Ayub Teaching Hospital, Abbottabad.

Bocca et al. in 2021 published a retrospective study regarding patients with acute subdural hematoma who were managed surgically [15]. Their results were quite alarming as more than half of the patients died and patients who were more than 75 years old had 100% mortality even after surgical management. Young age emerged to be significantly related to good outcomes in their study. Our results were quite similar to that of Bocca et al., as 58.4% of our patients had poor outcomes and old age was associated with poor outcomes in our data set.

Sharma et al. published American data regarding predictors of poor outcomes in patients after surgery of subdural hematoma. It was revealed that surgical evacuation of subdural hematomas overall was associated with favorable outcomes and age, female gender, focal neurologic deficit, and neuropsychiatric symptoms predicted 90-day functional outcomes [16]. More than half of the patients managed for acute subdural hematoma at our neurosurgical unit had poor outcomes. Advancing age, low GCS score at presentation, and use of oral anticoagulation therapy emerged as risk factors for poor outcomes in our study participants.

Factors contributing to outcomes after severe traumatic brain injury due to acute subdural hematoma in patients admitted to National Hospital Abuja, Nigeria, was published in 2021 [17]. It was concluded that the rate of unfavorable outcomes in acute subdural hematoma was high. The Glasgow Coma Score at admission was an important predictor for outcomes in their study participants. A total of 41.6% of patients in our study had good outcomes and the GCS score at presentation was also the factor associated with poor outcomes in our study (p<0.001).

Thirty days’ mortality in patients of acute subdural hematoma was studied by Pastor et al. in 2021 in Romanian patients. They came up with the findings that mortality rate was around 46% in their sample population and low GCS score was statistically significantly associated with poor outcome. Our study results supported the findings generated by Pastor et al. Treating team should be aware of factors related to poor outcome and decision-making regarding various options should incorporate these factors [18].

Data from this study reveals that outcomes of evacuation surgery for acute subdural hematoma are not consistent and a large number of patients showed poor short-term outcomes. Patients with risk factors for poor outcomes should be dealt with more attention in order to make the outcomes of the procedure better.

Study limitations

Patients were not followed up for long therefore exact outcomes could not be determined. Effect modifying and confounding facts for outcome in our study participants were not controlled strictly therefore associations cannot be established with precision. All patients were not managed by the same neurosurgeon therefore chance of bias remains in design.

## Conclusions

More than half of the patients managed with surgical evacuation for acute subdural hematoma as per guidelines at our neurosurgical unit had poor outcomes according to Glasgow Outcome Scale. In this study advancing age (>60 years), low GCS score at presentation, and use of oral anticoagulation therapy emerged as significant risk factors for poor outcomes in participants.

## References

[1] Yadollahi M (2019). A study of mortality risk factors among trauma referrals to trauma center, Shiraz, Iran, 2017. Chin J Traumatol.

[2] Crupi R, Cordaro M, Cuzzocrea S, Impellizzeri D (2020). Management of traumatic brain injury: from present to future. Antioxidants (Basel).

[3] Alihonou T, Quenum K, Padonou A, Amossou F, Dossou F (2020). Neurosurgical emergencies at a tertiary referral center in a Sub-Saharan African Country. J Neurosci Rural Pract.

[4] Yatsushige H (2021). Surgical management of a post-traumatic intracranial hematoma. [Article in Japanese]. No Shinkei Geka.

[5] Lee JJ, Segar DJ, Morrison JF, Mangham WM, Lee S, Asaad WF (2018). Subdural hematoma as a major determinant of short-term outcomes in traumatic brain injury. J Neurosurg.

[6] Shin DS, Hwang SC (2020). Neurocritical management of traumatic acute subdural hematomas. Korean J Neurotrauma.

[7] Dijck JT, Essen TA, Dijkman MD, Mostert CQ, Polinder S, Peul WC, de Ruiter GC (2019). Functional and patient-reported outcome versus in-hospital costs after traumatic acute subdural hematoma (t-ASDH): a neurosurgical paradox?. Acta Neurochir (Wien).

[8] Tverdal C, Aarhus M, Rønning P, Skaansar O, Skogen K, Andelic N, Helseth E (2022). Incidence of emergency neurosurgical TBI procedures: a population-based study. BMC Emerg Med.

[9] Lavrador JP, Teixeira JC, Oliveira E, Simão D, Santos MM, Simas N (2018). Acute subdural hematoma evacuation: predictive factors of outcome. Asian J Neurosurg.

[10] Asif M, Rehman WA, Sarwar S, Younas H, Younas A (2021). Acute traumatic subdural hematoma: series of thirty cases. Pak J Neurol Surg.

[11] Trevisi G, Sturiale CL, Scerrati A (2020). Acute subdural hematoma in the elderly: outcome analysis in a retrospective multicentric series of 213 patients. Neurosurg Focus.

[12] Ha JH, Park JH, Jeong JH, Im SB, Hwang SC (2018). Expanding subdural hematomas in the subacute stage and treatment via catheter drainage. Korean J Neurotrauma.

[13] Ruggeri AG, Cappelletti M, Tempestilli M, Fazzolari B, Delfini R (2022). Surgical management of acute subdural hematoma: a comparison between decompressive craniectomy and craniotomy on patients treated from 2010 to the present in a single center. J Neurosurg Sci.

[14] Yamal JM, Hannay HJ, Gopinath S, Aisiku IP, Benoit JS, Robertson CS (2019). Glasgow Outcome Scale measures and impact on analysis and results of a randomized clinical trial of severe traumatic brain injury. J Neurotrauma.

[15] Bocca LF, Lima JV, Suriano IC, Cavalheiro S, Rodrigues TP (2021). Traumatic acute subdural hematoma and coma: retrospective cohort of surgically treated patients. Surg Neurol Int.

[16] Sharma R, Rocha E, Pasi M, Lee H, Patel A, Singhal AB (2020). Subdural hematoma: predictors of outcome and a score to guide surgical decision-making. J Stroke Cerebrovasc Dis.

[17] Igbokwe KK, Ayogu OM, Onobun DE, Essiet EA, Ugwuanyi UC (2021). The outcomes of traumatic acute subdural hematoma in a tertiary center in Abuja, Nigeria. Cureus.

[18] Pastor IS, Dumbravă LP, Siserman C, Stan H, Para I, Florian IȘ (2021). Predictive factors of 30-day mortality in patients with traumatic subdural hematoma. Exp Ther Med.

